# A Distributed Air Index Based on Maximum Boundary Rectangle over Grid-Cells for Wireless Non-Flat Spatial Data Broadcast

**DOI:** 10.3390/s140610619

**Published:** 2014-06-17

**Authors:** Seokjin Im, JinTak Choi

**Affiliations:** 1 Department of Computer Engineering, Sungkyul University, 53, SungkyulDaehak-Ro, Manan-Gu, Anyang, Gyeonggi-Do 430-742, Korea; E-Mail: imseokjin@sungkyul.edu; 2 Department of Computer Engineering, Incheon National University, 119 Academy-Ro, Yeonsu-Gu, Incheon 406-772, Korea

**Keywords:** non-flat wireless data broadcast, distributed indexing scheme, spatial data, window query

## Abstract

In the pervasive computing environment using smart devices equipped with various sensors, a wireless data broadcasting system for spatial data items is a natural way to efficiently provide a location dependent information service, regardless of the number of clients. A non-flat wireless broadcast system can support the clients in accessing quickly their preferred data items by disseminating the preferred data items more frequently than regular data on the wireless channel. To efficiently support the processing of spatial window queries in a non-flat wireless data broadcasting system, we propose a distributed air index based on a maximum boundary rectangle (MaxBR) over grid-cells (abbreviated DAIM), which uses MaxBRs for filtering out hot data items on the wireless channel. Unlike the existing index that repeats regular data items in close proximity to hot items at same frequency as hot data items in a broadcast cycle, DAIM makes it possible to repeat only hot data items in a cycle and reduces the length of the broadcast cycle. Consequently, DAIM helps the clients access the desired items quickly, improves the access time, and reduces energy consumption. In addition, a MaxBR helps the clients decide whether they have to access regular data items or not. Simulation studies show the proposed DAIM outperforms existing schemes with respect to the access time and energy consumption.

## 1. Introduction

With the advent of smart mobile devices such as smart phones and tablets, which have processing capabilities almost as powerful as computers, and advances in wireless networks (e.g., the 4th generation mobile communication technology), the vision of pervasive computing has become real [1,2]. Smart devices equipped with various sensors and located in smart spaces become aware of mobile clients' contexts, such as physical location and provide them with various information services in the right place and at the right time [3–5]. For example, a mobile client in an emergency can search for the nearest hospital relative to its location on a road. Thus, context awareness is becoming more and more pervasive in our daily lives. In particular, context awareness of physical location is one of the key attributes for pervasive computing, which leverages various useful information services. In addition, the convenience provided and people's interest in the pervasive computing vision greatly enlarge the population of mobile clients.

On the road to making the pervasive computing vision become reality, via efficient information services using context awareness, scalability and location become two important challenges to overcome. That is why the number of mobile clients simultaneously demanding information related to current location is increasing tremendously. In the computing environment, a wireless data broadcasting system for spatial data items is a natural way to resolve the two challenges of scalability and location, because it can efficiently provide location dependent information services regardless of the number of clients [6]. A wireless data broadcasting system can effectively support access by a large number of mobile clients to information of interest because the system can simultaneously accommodate an arbitrary number of clients [7–10].

In a wireless broadcast system, a server periodically disseminates a set of data items through a wireless channel. Then, the clients tune in to the channel in energy-consuming mode (active mode) and download the desired items. Thus, the system provides high scalability for all clients downloading data of interest, independent of each other, without the server overload [11,12].

The system introduces an air indexing scheme to support the clients to energy-efficiently access data on the channel [9,13,14]. An air index holds information about the time when data items are broadcast on the wireless channel. The index is disseminated with data items on the channel. After tuning in to the channel, the clients can determine the time the desired data items will appear on the channel using the index, which they meet first, and switch to the doze mode (energy-saving mode) until that time. Then, the clients wake up at the predetermined time using the index, change to the active mode, and download the items from the wireless channel. Accordingly, the clients selectively listen to only the desired items. As one of the performance metrics for the system, the access time is measured by the time elapsed from starting the process of a given query to downloading all data items, the answer to the query. The tuning time is the time when a client stays in the active mode during the access time in order to download indexes and data items from the channel [15].

The broadcasting system can take one of two approaches (flat data broadcasting and non-flat data broadcasting) according to considering clients' preference to data items or not [9,10,14,16,17]. In a flat wireless data broadcasting approach, the server disseminates all data items with the same frequency in a broadcast cycle. On the other hand, in a non-flat data broadcast approach, the server broadcasts popular data items, called hot data items, more frequently than the regular data in a cycle. In order to select hot data items in the non-flat approach, the clients send their data preferences to the server through a low-bandwidth uplink channel [10,18,19]. Then the server chooses the preferred data items as hot data and broadcasts them more frequently than regular data through a high-bandwidth downlink channel. When the clients show the access patterns weighted to hot data items, a non-flat approach reduces the average access time because the clients can access hot items more quickly than regular data on the channel.

**Figure 1.**
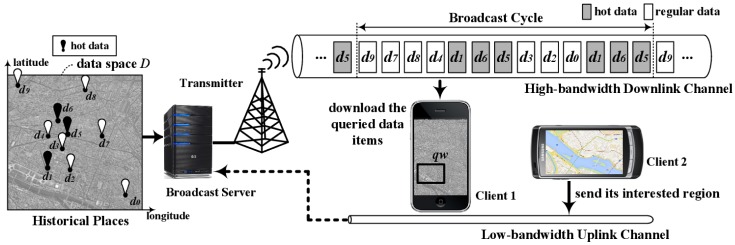
A system for the non-flat broadcast of spatial data.

A system that broadcasts spatial data items efficiently provides location dependent information services (LDIS) [20]. Here, spatial data items have geographic information about features in a data space (a two-dimensional geographic area), such as data on shopping malls in a city. Figure 1 shows a system broadcasting historic places in a metropolitan city like New York using the non-flat approach. In the system, the clients can find famous historic places within a given query window, like *qw* in Client1 of Figure 1, over a data space by listening to the channel. For example, a client can find historic places in the city within one square kilometer centered at its current location.

To help the clients efficiently access spatial data items of interest in an LDIS via wireless data broadcasts, air indexing schemes have been proposed for both flat and non-flat wireless data broadcasts. In particular, a grid-based distributed index (GDIN) in a non-flat spatial data broadcast was proposed to help the clients access desired data items when clients' data access patterns are skewed to hot data items [10]. GDIN, however, causes the clients to wait for their desired data items for a long time due to the lengthened broadcast cycle that results from the server's dissemination of regular items near hot data items as frequently as hot data items.

In this paper, we propose a distributed air index based on a maximum boundary rectangle (MaxBR) over grid-cells (abbreviated DAIM) for non-flat spatial data broadcasting to efficiently support processing window queries when clients' data access patterns are skewed. DAIM aims to improve the access time and to reduce energy consumption by more frequently replicating hot data only, unlike GDIN. To reach that goal, DAIM uses a grid partition of the data space and then broadcasts data items in those cells. Unlike GDIN, DAIM avoids repeating regular data in close proximity to hot data with the same frequency as hot items, by separating hot data and regular data with MaxBRs over a grid partition. A MaxBR is a maximum rectangle containing only hot data items in a grid-cell.

For the non-flat broadcast, DAIM broadcasts hot data items within MaxBRs in grid-cells more frequently in a broadcast cycle and broadcasts regular data items only once in the cycle. Thus, DAIM shortens the length of the broadcast cycle by more frequently replicating only hot data items. In addition, a MaxBR helps the clients decide whether they have to access regular data items or not. The query window contained in a MaxBR means that the client does not have to access regular data items. Additionally, DAIM enables the clients to access their desired hot data items quickly by providing links to cells on the wireless channel. In order to take into consideration the clients access the channel linearly, DAIM adopts a linear table structure.

The rest of the paper is organized as follows. We summarize air indexing schemes in flat and non-flat broadcast schemes under Related Works in Section 2. We present the proposed DAIM scheme in Section 3, and evaluate its performance by comparing it to existing schemes in Section 4. Finally, we conclude the paper in Section 5.

## 2. Related Works

### 2.1. Air Index Allocation Schemes and Data Search

Air indexing schemes let the clients listen selectively to queried data items in an energy-efficient way by filtering out and downloading the items from the wireless channel [21].

#### 2.1.1. Air Index Allocation Schemes

An air index can be allocated to data items on the wireless channel in two schemes, a (1, *m*) index scheme and a distributed index scheme. The (1, *m*) index allocation scheme makes the index for all of the data items allocated *m* times preceding every (1/*m*) fraction of data items on the wireless channel [9,22,23]. Under the scheme, the clients can obtain the information on all of the data items from an index on the channel. However, it increases the length of the broadcast cycle because it repeats the index for all data items. In addition, it takes a long time to meet the index after tuning in to the channel because of the lengthened interval between index broadcasts.

The distributed index allocation scheme divides all data items into fractions, and then allocates the index for the data items in each fraction ahead of its data fraction on the channel [14,24]. The scheme has a shorter broadcast cycle than the former scheme because it broadcasts indexes for fractions of data, not for all data items. It also takes less time to meet the index after tuning in to the channel than under the (1, *m*) index scheme because of the shortened interval between indexes. Thus, the scheme has the advantage of improving the access time that is affected by the length of the broadcast cycle, and lessens the time to reach the first index after tuning in to the channel. In this scheme, it is important to provide link information to indexes on the channel to support that the clients efficiently process queries by accessing indexes one after another on the channel.

The proposed DAIM applies the distributed index allocation scheme in order to improve the access time. For the allocation scheme, we partition data fractions with an *n* × *n* grid partition of the data space.

#### 2.1.2. Data Search

Using the air index disseminated on the channel, the clients can process a given query in the following manner. A client determines the time the index appears on the broadcasting channel using the first bucket it meets after tuning in to the channel. Here, the bucket is the smallest logical access unit. Then, it switches to the doze mode until the predetermined time, waiting for the index. The probe wait is the time duration from tuning in to the channel to accessing the index.

The client switches to the active mode the moment the index appears on the channel. Then, it downloads the index and searches it for the queried data items. Finally, it filters out the items and determines the time the data items will appear on the channel. The client switches to the doze mode by the predetermined time. It then awakens and downloads the data items. The bcast (broadcast) wait is the time duration from accessing the index to downloading all queried data items from the channel.

### 2.2. Spatial Data Broadcasting Schemes

#### 2.2.1. Flat Broadcasting

For broadcasting spatial data to support an LDIS, various indexing schemes have been proposed for the clients to process spatial queries. Under a flat broadcast with spatial data, Hilbert Curve Index (HCI) [9] and Distributed Spatial Index (DSI) [17] were proposed using a Hilbert Curve (HC). With these two schemes, the clients have to use excessive energy for given queries because the items are extracted after listening to too many candidates decided with an HC value for each item, not with its real coordinates.

The authors proposed Cell-based Distributed Index (CEDI) for energy-efficient query processing, in which the clients listen to only their desired data items by extracting them using their real coordinates, unlike the two schemes based on an HC [14].

#### 2.2.2. Nonflat Broadcasting

**Figure 2.**
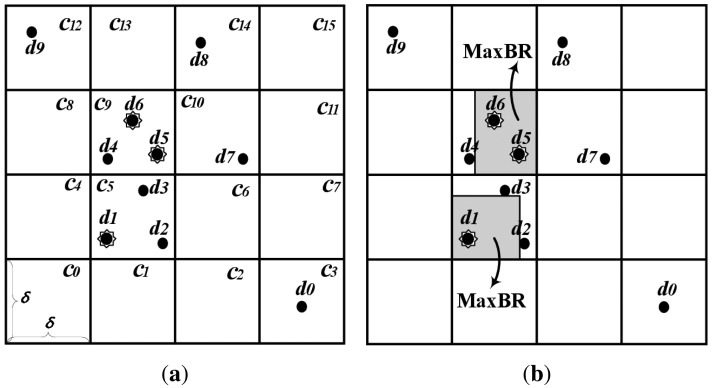
Grid partition and MaxBRs. (**a**) 4 × 4 grid for Figure 3a; (**b**) MaxBR.

For a non-flat broadcast with spatial data, the authors proposed an indexing scheme, a Grid-based Distributed Index for Non-flat broadcast (GDIN), for window query processing [10]. GDIN uses regular grid cells to index data items and defines a hot cell as a cell containing one or more hot data items. For the non-flat approach, GDIN broadcasts hot cells more frequently than regular cells that contain only regular data items. GDIN causes the access time deteriorated with the lengthened broadcast cycle because regular data items contained within hot cells are broadcast with the same frequency as hot data items. For example, regular data items, *d*_2_, *d*_3_ and *d*_4_ within cell *c*_5_ and *c*_9_ in Figure 2a, are replicated as frequently as hot data items. Also, if all cells contain one or more hot data items, GDIN works as a flat broadcast, not a non-flat broadcast.

In this paper, we set out to solve the problem with GDIN that causes the deteriorated access time from repeating regular data items as frequently as hot data items. As a solution, we adopt MaxBRs over a grid partition to separate hot data items and regular data in the data space.

## 3. Proposed Indexing Scheme

**Figure 3.**
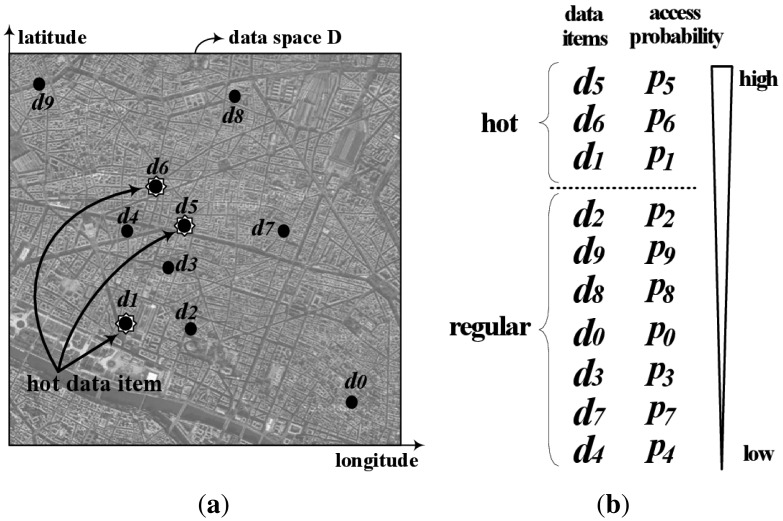
Data space *D* and deciding hot data items. (**a**) Data space *D*; (**b**) Decision of hot data items.

We consider a non-flat broadcasting system with a high-bandwidth downlink channel and a low-bandwidth uplink channel as shown in Figure 1. The server disseminates over the downlink channel a set of spatial data items, {*d*_0_, *d*_1_, … , *d_N_*_− 1_}, in two-dimensional data space *D* defined with longitude and latitude as the axes, as shown in Figure 3a.

For the non-flat data broadcast, the clients send their area of interest *A_int_* over *D* through the uplink channel. Then, access probability *p_i_* of data item *d_i_* is calculated as follows:

(1) $$p_{i}=f_{i}/(\sum_{j=1}^{N}f_{j})$$

Here, *f_i_* is the number of *A_int_* overlapped with *d_i_*. Then, the server chooses the first [*N* · *R_h_*] items as hot data items, listing *N* items in decreasing order of *p_i_*. Here, *R_h_* is a ratio to determine the number of hot items. For example, Figure 3a shows the locations of 10 historic places with dots. Figure 3b shows the list of 10 items with their access probability in decreasing order. The first three items, *d*_5_, *d*_6_ and *d*_1_, are selected as hot data items in *R_h_* = 0.28.

To organize the proposed DAIM, we partition the data space *D* with an *n* × *n* grid as shown in Figure 2a. Cells are named in row major order, *c_k_* (0 ≤ *k* < *n*^2^). Then, a MaxBR is defined as a maximum rectangle containing only hot items in a grid-cell with the coordinates of the lower-left and upper-right corner. For example, Figure 2b shows two MaxBRs in *c*_5_ and *c*_9_.

### 3.1. Index Structure

The proposed scheme DAIM has three kinds of indexes: a global index (*GI*), a MaxBR index (*MI*), and a cell index (*CI*). The *GI* keeps information on the MaxBRs and all cells with data items. The *MI* for each MaxBR holds the information on hot data items in the MaxBR. The *CI* for each cell with data items holds information on regular data items within it, along with the link to the global index appearing next on the wireless channel.

The *GI* is constructed as follows:

(2) GI=〈MT,RT〉

- *MT* (MaxBR Table): {< *c_k_, MaxBR, t_m_* > | 0 ≤ *k* < *n*^2^}, where *c_k_* is a cell with hot data items; *MaxBR* is defined in *c_k_* with the coordinates of the lower-left corner and upper-right corner, (*LL_x_, LL_y_*) and (*UR_x_, UR_y_*); * t_m_* is the broadcasting time for the *MI*.
- *RT* (Regular Table): {< *c_k_, t_k_* > | 0 ≤ *k* < *n*^2^}, where *c_k_* is a cell with regular data items and *t_k_* is the broadcasting time for *CI_k_* of *c_k_*.

The *GI* lets the clients decide MaxBRs and cells to access for a given query and broadcasting times for them. The *GI* also provides the clients with links to the MaxBRs and cells in order to access efficiently.

*MI* and *CI_k_* are constructed as follows:

(3) $$\{\begin{array}{ll} MI & =\langle t_{gi},COT\rangle \\ CI_{k} & =\langle t_{gi},COT\rangle ,\text{for}0\leq k<n^{2} \end{array}$$

- *t_gi_* is the time when the *GI* appears right after the *MI* or *CI_k_* on the wireless channel.
- *COT* (Coordinate Table): {< (*d_x_, d_y_*), *t_d_* >} Here, (*d_x_, d_y_*) represents the coordinates of data item, *d*, belonging to the MaxBR or *c_k_* and *t_d_* is the time when *d* is broadcast on the wireless channel.

*COT* enables the clients to extract the items contained within a given query window from data items in a MaxBR or *c_k_* before downloading the queried data items from the channel.

Algorithm 1 depicts the procedure of finding MaxBRs in a cell, *c_k_*, to generate the content of *MT* in *GI* and *COT* in *MI*. The algorithm has two sets of data items as the input, *S_hot_* of hot data items and *S_reg_* of regular ones in the cell and returns a set of entries of a MaxBR and a queue holding hot data items containing the MaxBR. The algorithm runs in the way that a MaxBR for a hot data item in *S_hot_* are determined first with all items in *S_reg_*, and then all hot items in *S_hot_* contained in the determined MaxBR are removed from *S_hot_* and enqueued to *Queue* for the MaxBR. The algorithm runs for the rest of hot items in *S_hot_* in the same way until *S_hot_* = *ϕ*.


**Algorithm 1***Finding MaxBR*
**Input**: *S_hot_* (a set of coordinates of hot data items in *c_k_*), *S_reg_* (a set of coordinates of regular ones in *c_k_*)**Output:***Result*, a set of entries <*c_k_*, (*LL_x_, LL_y_*),(*UR_x_, UR_y_*), *Queue*>1: **while** (*S_hot_* ≠ *ϕ*)2:  initialize (*LL_x_, LL_y_*) and (*UR_x_, UR_y_*) with the coordinates of the lower-left point and upper-right point of *c_k_*;3:   (*hd_x_, hd_y_*) ← (*d_x_, d_y_*) in *S_hot_*4:  **foreach** (*d_x_,d_y_*) in *S_reg_*5:   **if** ((*LL_x_* <= *d_x_* <= *UR_x_*)&&(*LL_y_* <= *d_y_* <= *UR_y_*))6:    **if** (*hd_x_* < *d_x_*)7:     (*p*1*_x_, p*1*_y_*) ← (*LL_x_, LL_y_*); (*p*2*_x_, p*2*_y_*) ← (*d_x_, UR_y_*);8:    **else**9:     (*p*1*_x_, p*1*_y_*) ← (*d_x_, LL_y_*); (*p*2*_x_, p*2*_y_*) ← (*UR_x_,UR_y_*);10:    **end if**11:    **if** (*hd_y_* < *d_y_*)12:    (*p*3*_x_, p*3*_y_*) ← (*LL_x_, LL_y_*); (*p*4*_x_, p*4*_y_*) ← (*UR_x_, d_y_*);13:    **else**14:    (*p*3*_x_, p*3*_y_*) ← (*LL_x_, d_y_*); (*p*4*_x_, p*4*_y_*) ← (*UR_x_, UR_y_*);15:    **end if**16:  **end if**17:  Area1 ← the area of the rectangle defined with (*p*1*_x_, p*1*_y_*) and (*p*2*_x_, p*2*_y_*)18:  Area2 ← the area of the rectangle defined with (*p*3*_x_, p*3*_y_*) and (*p*4*_x_, p*4*_y_*)19:  **if** (Area1 > Area2)20:   (*LL_x_, LL_y_*) ← (*p*1*_x_, p*1*_y_*); (*UR_x_, UR_y_*) ← (*p*2*_x_, p*2*_y_*);21:  **else**22:   (*LL_x_, LL_y_*) ← (*p*3*_x_, p*3*_y_*); (*UR_x_, UR_y_*) ← (*p*4*_x_, p*4*_y_*);23:  **end if**24: **end foreach**25: create a *Queue*;26: **foreach** (*d_x_, d_y_*) in *S_hot_*27:  **if** MaxBR defined with (*LL_x_, LL_y_*) and (*UR_x_, UR_y_*) ⊃ (*d_x_, d_y_*)28:   enqueue (*d_x_, d_y_*) to *Queue*;29:  **end if**30: **end foreach**31: delete all items in **Queue** from *S_hot_*;32: add a tuple < *c_k_*, (*LL_x_, LL_y_*),(*UR_x_, UR_y_*), *Queue* > to *Result*;33:**end while**34:**return**
*Result*;


In order to determine the MaxBR for a hot data item in *S_hot_*, the MaxBR is set initially to the lower-left point and upper-right point of *c_k_* in line 2. From line 4 to 24, the MaxBR is renewed with the maximum rectangle among the two rectangles containing the hot data item, which are divided from the current MaxBR by the coordinates of a regular data item in *S_reg_*. Then, from line 25 to 31, all hot data items in *S_hot_* contained within the renewed MaxBR are enqueued into *Queue* and removed from *S_hot_*. In line 32, an entry < *c_k_*, (*LL_x_, LL_y_*), (*UR_x_, UR_y_*), *Queue* > is added to Result. Here, *c_k_*, (*LL_x_, LL_y_*), and (*UR_x_, UR_y_*) are used for generating the content of *MT* in *GI* and Queue is used for *COT* in *MI* and *CI*.

The execution time of Algorithm 1 is proportional to |*S_hot_*‖*S_reg_*| by the while-loop and the foreach-loop in the algorithm. Here |*S_hot_*| and |*S_reg_*| are the the size of *S_hot_* and *S_reg_*, respectively. Therefore, the time complexity of the algorithm is *O*(*|S_hot_*‖*S_reg_*|).

### 3.2. Channel Structure

With the proposed DAIM, the wireless broadcast channel is basically organized such that broadcasting of the data items and indexes is in row major order for the grid partition. To disseminate hot data items within MaxBRs more frequently as the non-flat approach, the *GI*, the *MI*, and hot data items are broadcast ahead of every row having data items on the channel. For a row, all cells having data items in the row are broadcast in order of increasing cell number. For a cell, the *CI* for it precedes all data items in it.

**Figure 4.**
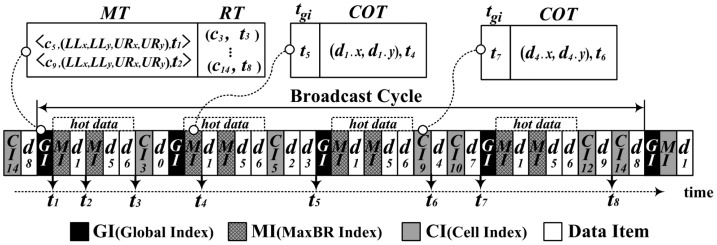
The wireless channel structure with DAIM.

Figure 4, for example, depicts the structure of the wireless broadcast channel for the grid partition shown in Figure 2. MaxBRs for hot data items are determined in *c*_5_ and *c*_9_. The cells containing regular data items are *c*_3_ in the first row, *c*_5_ in the second, *c*_9_ and *c*_10_ in the third, and *c*_12_ and *c*_14_ in the fourth. The server organizes the *GI* with the *MT* for hot data items and the *RT* for regular data items, the *MI* for each MaxBR, and the *CI* for each cell containing regular data items. Figure 4 shows the *GI*, the *MI* for the MaxBR in *c*_5_, and the *CI* for *c*_9_, respectively. Then, the server disseminates the *GI, MI*, and hot data items on the wireless channel by interleaving the cells in row major order. As shown in Figure 4, the *GI, MI*, and hot data items are interleaved with cells in each row having regular data items.

Thus, DAIM enables multiple repetitions of only hot data in a broadcast cycle, unlike GDIN, and boosts the performance with respect to the access time by reducing the length of the broadcast cycle while maintaining the advantages of a non-flat approach.

### 3.3. Window Query Processing

With DAIM, a client processes its own window query defined with query window *qw*, as shown in Figure 1, in the following three steps.

- *Step* 1. the client determines *Q*, which is a set of cells overlapped with *qw* as follows:
  (4) $$Q=\left\{c_{i}|i=a\cdot n+b\right\}$$
  Here, ⌊*LL_y_/δ*⌋ ≤ *a* ≤ ⌊*UR_y_/δ*⌋ and ⌊*LL_x_/δ*⌋ ≤ *b* ≤ ⌊*UR_x_/δ*⌋. (*LL_x_, LL_y_*) and (*UR_x_, UR_y_*) are the coordinates of the lower-left and upper-right corner of *qw* and *δ* is the length of the side of a cell.
- *Step* 2. This step begins with access to the *GI* on the channel. The client determines times to access the *MI* and the *CI* with entries for *MT* and *RT* of the *GI* as follows:
  $$\begin{array}{l} \{\begin{array}{l} \text{for each entry}<c_{k},\text{MaxBR},t_{m}>in\;MT\\ \;\text{if}\left(c_{k}\in Q\right)\\ \;\text{if}\left(\text{MaxBR}⊃qw\right)\\ \;\text{clear}\;\text{iQueue};\;\text{iQueue}\leftarrow t_{m};\;\text{break};\\ \;\text{if}\left(\left(\text{MaxBR}\cap qw\right)\neq \phi \right)\text{iQueue}\leftarrow t_{m}; \end{array}\\ \{\begin{array}{l} \text{for each entry}<c_{k},t_{k}>in\;RT\\ \;\text{if}\left(c_{k}\in Q\right)\text{iQueue}\leftarrow t_{k}; \end{array} \end{array}$$
  Here, *iQueue* is a queue that keeps the times in increasing order when the client accesses the *MI* or the *CI* on the channel to process the given query, *qw*.
- *Step* 3. After accessing the *MI* and *CI* using its broadcasting time in *iQueue*, the client extracts the times when data items contained within *qw* appear on the channel from the *COT* of the *MI* and *CI* as follows:
  $$\{\begin{array}{c} \text{for each entry}<\left(d_{x},d_{y}\right),t_{d}>\text{of COT}\\ \text{if}\left(\left(d_{x},d_{y}\right)\subset qw\right)\;\text{dQueue}\leftarrow t_{d}; \end{array}$$
  Here, *dQueue* is a queue keeping the times in increasing order when the data items belonging to *qw* are broadcast on the channel. Then, the client downloads the extracted data items from the channel at the times in *dQueue.*

Algorithm 2 shows the window query processing procedure under DAIM. The clients get their desired data items by the Steps 1, 2 and 3 mentioned above. Algorithms 3 and 4 show the procedures for the *doStep2WithGI*() function and *doStep3WithMIorCI*() function in lines 8 and 10 of Algorithm 2, respectively. They return the results of Step 2 and Step 3.

We analyze the time complexity of the three algorithms. To analyze the time complexity of Algorithm 2, we analyze the time complexity of Algorithms 3 and 4 first, because the algorithm is dependent on Algorithms 3 and 4, calling them to determine *iQueue* and *dQueue.* The execution time of Algorithm 3 is proportional to the number of entries of *MT* and *RT* in *GI*, because of the foreach-loop in the algorithm. In order to express the time complexity in the Big-O notation, we define the size of *GI*, |*GI*|, as follows:

(5) |GI|=max(|MT|,|RT|)

Here, |*MT*| and |*RT*| mean the number of entries of MT and RT, respectively. Function *max*() returns the maximum value of the values from the given input arguments. Therefore, the time complexity of Algorithm 3 is *O*(|*GI*|).


**Algorithm 2***WindowQuery*
**Input**: *qw* (a query window)**Output**: *Result*, a set of data items belonging to *qw*1: *Q* ← the cells overlapped with *qw*;// Step 12: tuning in on the wireless channel;3: *Index* ← the index encountered firstly after tuning-in;4: **if**(*Index* is *MI* or *CI*) **then**5:  access *GI* at *t_gi_* of *Index*;6:  *Index* ← *GI* accessed;7: **end if**8:  *iQueue* ← *doStep*2*WithGI*(*Index*);// Step 29: **for** all the indexes pointed by *iQueue***do**10:  *dQueue* ← *doStep*3*WithMIorCI*(*Index*) ;// Step 311:  *Result* ← all data items at the times in *dQueue*;12: **end for**13: **return***Result* ;



**Algorithm 3***doStep*2*WithGI*
**Input**: *Index* (*GI*)**Output**: *iQueue*, a queue keeping the broadcasting times for MaxBR and cells in *Q*1: **foreach** < *c_k_, MaxBR, t_m_* > in *MT***do**2:  **if***c_k_* ∈ Q3:   **if**(*MaxBR* ⊃*qw*) **then**4:    clear *iQueue*; *iQueue* ← *t_m_* ; break;5:   **end if**6:   **if**((*MaxBR* ∩ *qw) ≠ ϕ*)) **then**7:    *iQueue* ← *t_m_* ;8:   **end if**9:  **end if**10: **end foreach**11: **foreach** < *c_k_, t_k_* > in *RT***do**12:   **if**(*c_k_* ∈ *Q*) **then**13:    *iQueue* ← *t_k_* ;14:   **end if**15: **end foreach**16: **return***iQueue* ;



**Algorithm 4***doStep*3*WithMIorCI*
**Input**: *Index* (*MI* or *CI*), *qw* (a query window)**Output**: *dpQueue*, a queue keeping the broadcasting times for data items1: **foreach** < (*d_x_, d_y_*), *t_d_* > in *COT***do**2:  **if**(*qw* ⊃ (*d_x_, d_y_*)) **then**3:   *dQueue* ← *t_d_* ;4:  **end if**5: **end foreach**6: **return***dQueue* ;


The execution time of Algorithm 4 is proportional to the number of entries of *COT* in *MI* or *CI*, because of the foreach-loop in the algorithm. Using |*COT*|, the number of entries of *COT*, the time complexity of the algorithm is given *O*(|*COT*|) in the Big-O notation.

The execution time of Algorithm 2 is determined by the for-loop on *iQueue* in the algorithm. Here, Algorithm 3 returns *iQueue* and Algorithm 4 is called in each iteration of the loop. Therefore, the time complexity of the algorithm is given *O*(|*GI*‖*COT*|) in the Big-O notation, using the time complexity of Algorithm 3 and Algorithm 4.

**Figure 5.**
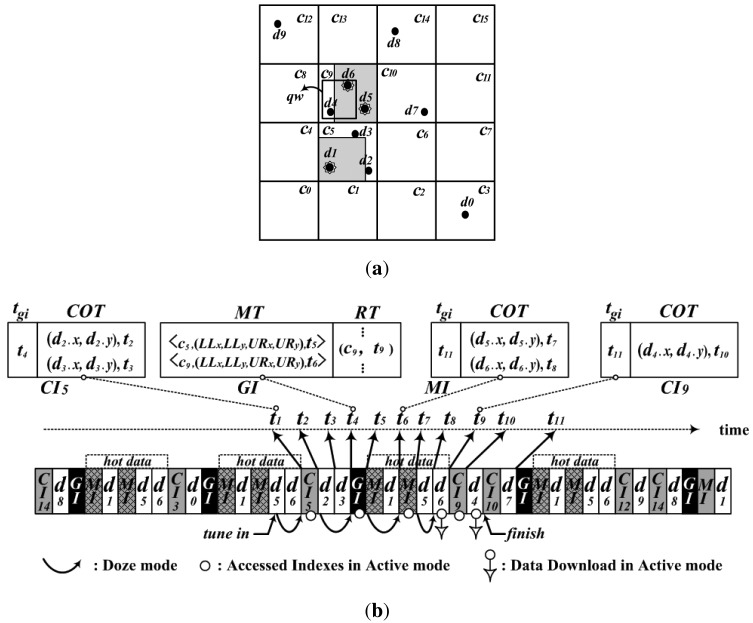
An example for query processing. (**a**) An Example Query; (**b**) The procedure for processing the example query.

Figure 5 shows an example of processing a window query with the proposed DAIM. The given window query, *qw*, is shown in Figure 5a. Figure 5b shows the procedure whereby a client processes *qw* on the wireless channel in a case where the client tunes in the channel in the middle of broadcasting *d_5_*. First of all, the client sets *Q* to {*c*_9_} as mentioned in Step 1, and tunes in the channel as described at line 2 of Algorithm 2. Then, the client accesses *CI*_5_ at *t*_1_ using the time pointer to the first index encountered, which is kept in the bucket header of the first complete bucket after tuning in. As described from lines 4 to 7 of Algorithm 2, the client accesses *GI* at *t*_4_ using *t_gi_* of *CI*_5_ and determines *iQueue*, as mentioned in line 8 of Algorithm 2. In the process for *iQueue*, the client inserts *t*_6_ into *iQueue* with *MT* of the accessed *GI* via lines 1 to 10 of Algorithm 3. Also, the client inserts *t*_9_ into *iQueue* with *RT* of the *GI* via lines 11 to 15 of Algorithm 3. Using the time pointers in *iQueue*, the client accesses the MI and *CI*_9_ sequentially, as described from lines 9 to 12 of Algorithm 2, and downloads data items contained within *qw* via Algorithm 4. After accessing the *MI* at *t*_6_, the client enqueues *t*_8_ to *dQueue* by using the *COT* of the accessed *MI* according to Algorithm 4 and downloads *d*_6_ at *t*_8_. Then, the client accesses *CI*_9_ and inserts *t*_10_ into dQueue just as it did with the *MI*. Last, the client downloads *d*_4_ at *t*_10_ and finishes processing the given query *qw*.

## 4. Performance Evaluation

We evaluate the performance through simulations of the proposed DAIM with respect to the access time and tuning time, and evaluate energy consumption during the processing of given window queries for practical implications. We demonstrate the efficiency of the proposed scheme by comparing DAIM against GDIN in non-flat spatial broadcasting and against HCI, DSI, and CEDI in flat spatial data broadcasting.

### 4.1. Simulation Environment

For evaluation of the performance through simulations of DAIM, we implemented a testbed using the discrete time simulation package SimJava [25]. The testbed consists of one broadcast server, 100 clients, a high-bandwidth downlink channel of 1 Mbps and a low-bandwidth uplink channel of 32 Kbps. The clients send regions of interest to the server via the uplink channel, and the server selects hot data items and broadcasts data items in the non-flat broadcast manner, as mentioned previously. For the simulations, we use a real skewed dataset of 5922 cities in Greece, including geographic locations for the cities by longitude and latitude [26].

The simulation parameters are as follows: the size of one data item is 1024 bytes; the size of a bucket as the smallest logical access unit changes from 64 to 512 bytes and the default bucket size is 64 bytes; the size of the bucket header, keeping the time pointer to the next index table, sets 8 bytes; the sizes of the cell identifier, the coordinates of a data item, and the time pointer are all set to 8 bytes; *R_qw_*, the ratio of one side of the query window to one side of the test area, is set from 0.02 to 0.1; *H_r_*, the ratio of hot data items to all broadcast data items, is set from 0.05 to 0.2; *N_q_*, the number of window queries issued by a client for one simulation condition, is 100, 000; and *R_hot_*, the portion of accessing hot data items of *N_q_*, is set from 0.6 to 1.0. To partition a data space, we set *n* from 8 to 64 for the proposed DAIM, GDIN, and CEDI, and set 16 as the default value of n. For each simulation condition, we measure the average access time and tuning time over *N_q_* queries in buckets.

Here, we use various bucket sizes for the evaluation because the size can affect the performances, *i.e.*, the access time, the tuning time, and the energy consumption. According to the bucket size, a data item is divided into the different number of buckets. That changes the size of the data item on the channel. The changed data size on the channel affects the performances. For example, when the bucket size is 64 with the bucket header size 8 bytes, a data item of 1024 bytes is divided into 19 buckets (converted into 1216 bytes). Thus, the size of the data item increases by 192 bytes on the channel. When the bucket size is 512 with the bucket header size 8 bytes, the data item is divided into 3 buckets (converted into 1536 bytes). The size of the data item on the channel by the bucket size 512 bytes increases much more than that by the bucket size of 64 bytes. Thus, as the bucket size increases, the number of buckets for a data item decreases, while the size of the data item in bytes on the channel increases. The increased data size by organizing buckets affects the access time of the clients by changing the length of a broadcast cycle and affects the tuning time by changing the data size for the clients to listen. Also, the energy consumption is affected by the changes in the access time and tuning time.

For fair comparisons, we also adopt the simulation parameters mentioned above for HCI and DSI. Especially for HCI, we set *m*, the number of replication of index information on the wireless channel, to 7, the value minimizing the access time of HCI used in [9]. For the same reason, the exponential base of DSI is set to 2.

### 4.2. Comparison of the Access Time

To show the effectiveness of the proposed DAIM, we compare it to GDIN for non-flat broadcast and to HCI, DSI, and CEDI for flat broadcast with respect to the access time. We evaluate the access times of the indexing schemes by varying *R_hot_, R_qw_*, the bucket size, *n*, and *H_r_*.

**Figure 6.**
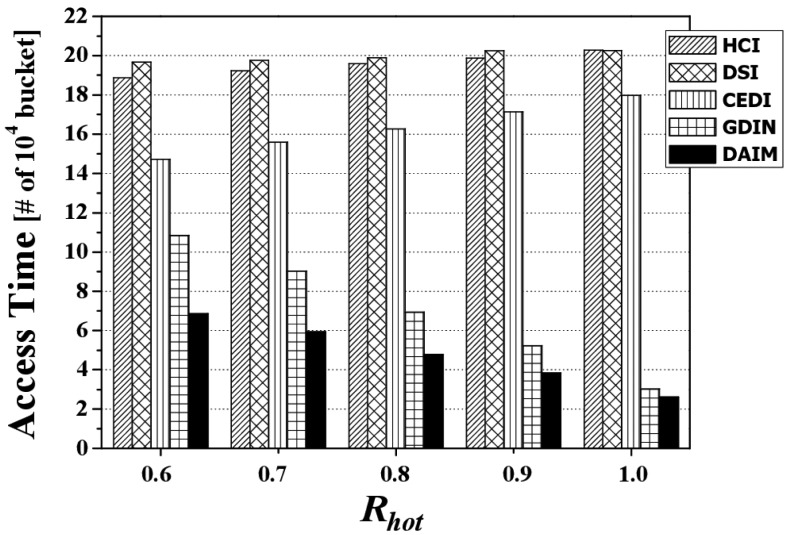
The comparison of the access time over various *R_hot_*.

Figure 6 depicts the access time by various *R_hot_* values when *R_qw_* is 0.06 and both bucket size and *n* are set to the default values. It reveals that the proposed DAIM outperforms GDIN as well as HCI, DSI, and CEDI when the clients access hot data items more frequently (*R_hot_* increases). This results from the shortened length of a broadcast cycle by replicating only hot data in a broadcast cycle, unlike GDIN, in which regular items within hot cells are replicated in a cycle as frequently as hot data items. When *R_hot_* is 0.9, the access time of DAIM is about 71% of that for GDIN.

Figure 6 shows the difference of the access time between GDIN and DAIM is reduced with *R_hot_* = 1.0 as compared to that with *R_hot_* = 0.8. It is because the access time depends on the period between a data item and the same data item appearing next on the wireless channel. When the clients access only hot data items with *R_hot_* = 1.0, the access time is affected by only the period for hot data items. When the clients access hot data items and regular ones with *R_hot_* = 0.8, the access time is affected dominantly by the period for regular items rather than that for hot data items because the period for regular items is much longer than that for hot data items. Accordingly, the difference of the access time between GDIN and DAIM with *R_hot_* = 1.0 and *R_hot_* = 0.8 depends on the difference of the period for hot items and regular items between GDIN and DAIM. Therefore, the difference of the access time between GDIN and DAIM results from the longer difference of the period for regular data items than that for hot data items between GDIN and DAIM.

**Figure 7.**
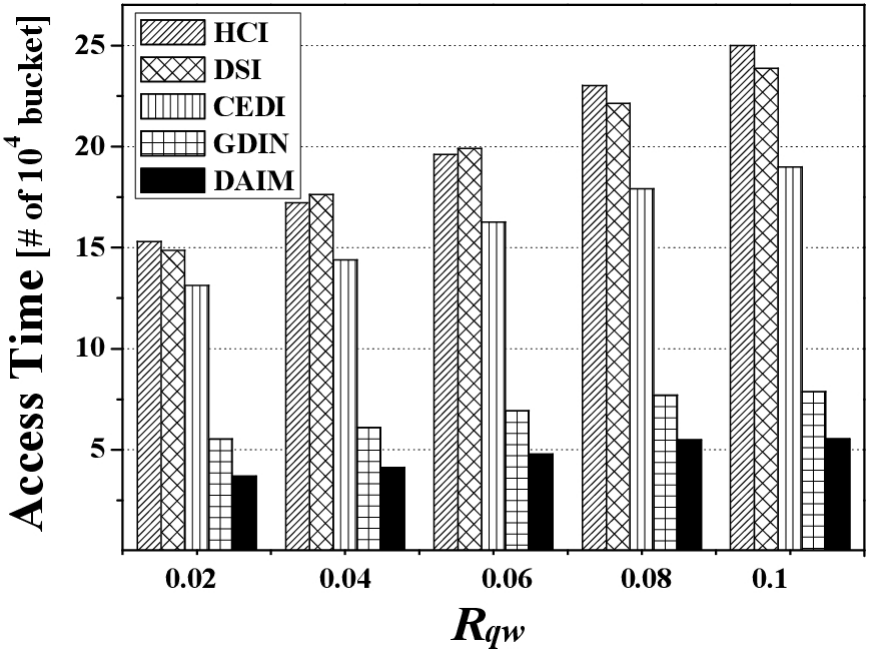
The comparison of the access time over various *R_qw_*.

Figure 7 depicts the access time by various *R_qw_* values when *R_hot_* is 0.8. Overall, the access time for each indexing scheme increases along with *R_qw_* because the larger *R_qw_* is, the more data items the clients have to access. The figure reveals that DAIM enables the clients to quickly access their desired data items with various sizes of window queries. Especially, with increased sizes of window queries, DAIM outperforms GDIN. Thus, the proposed DAIM helps the clients efficiently process window queries on air.

**Figure 8.**
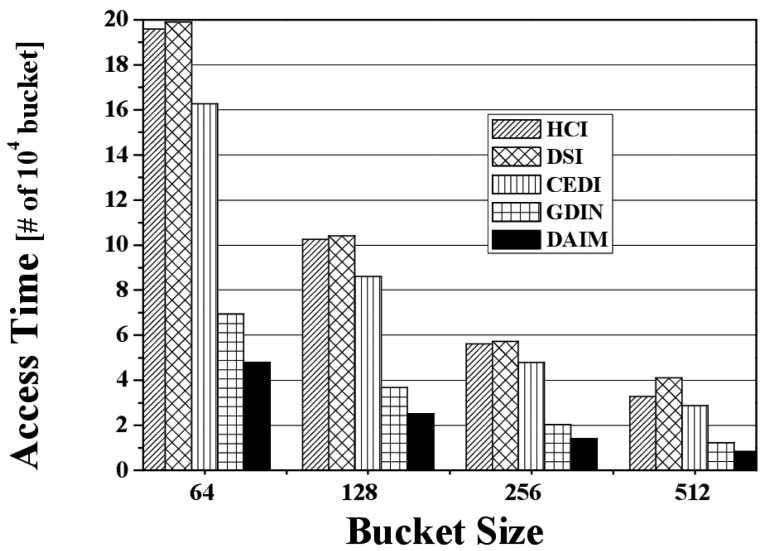
The comparison of the access time over various bucket sizes.

Figure 8 shows the change in the access time when data items and indexes are disseminated at various bucket sizes. The figure depicts the access time for various bucket sizes when *R_qw_* and *R_hot_* are 0.06 and 0.8, respectively. We measure the access time in buckets because the bucket is the smallest logical unit in which the clients access data items. As the bucket size increases, the access time decreases. That is because as the bucket size increases, the number of buckets decreases. The access time for DAIM is shorter than GDIN in the non-flat scheme, as well as HCI, DSI, and CEDI in the flat scheme. This demonstrates that DAIM helps the clients access the desired data items quickly at all bucket sizes.

**Figure 9.**
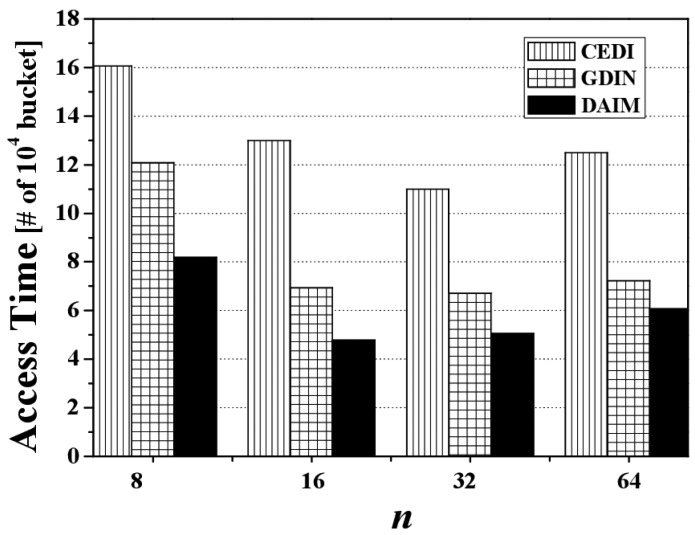
The comparison of the access time over various n.

Next, Figure 9 shows the effect of changing n, the grid partition, on the access time for CEDI, GDIN, and DAIM when *R_qw_* and *R_hot_* are 0.06 and 0.8, respectively. Figure 9 reveals that DAIM outperforms GDIN and CEDI over various values of *n*. At *n* = 64, the access time under DAIM is about 68% and 51% of GDIN and CEDI, respectively.

The value of *n* affects the access time by adjusting the length of the entire broadcast cycle and the probe wait that the time duration until the access to the first index after tuning in to the channel in the three schemes. The length of the broadcast cycle is affected by the number of the index tables and the probe wait is affected by the period between index tables on the wireless channel. At smaller *n*, the length of the broadcast cycle increases a little because the number of the index tables is small. The probe wait is large because the period between the index tables is large. Therefore, the access time increases by the effect of the large probe wait at smaller *n*. At larger *n*, the length of the broadcast cycle increases much longer because the number of the index tables increases. The probe wait is small because the period between the index tables is shrunken much. Therefore, the access time increases by the effect of increasing the length of the broadcast cycle at larger *n*.

**Figure 10.**
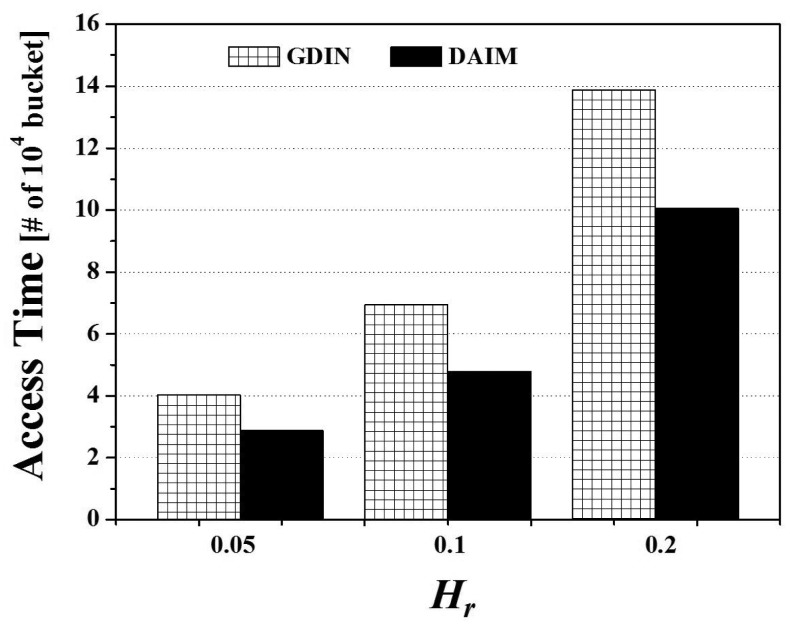
The comparison of the access time over various *H_r_*.

Finally, Figure 10 shows the effects of the change of *H_r_*, the ratio of hot data items, on the access time for the two non-flat broadcasting schemes, GDIN, and DAIM, when *R_qw_* and *R_hot_* are 0.06 and 0.8, respectively. The figure illustrates that the different distribution of the access probabilities of data items affects the access time. Figure 10 reveals that DAIM outperforms GDIN over various values of *H_r_*. As *H_r_* increases, the access time increases. It is because the length of the broadcast cycle increases by increasing the number of hot data items.

### 4.3. Comparison of the Tuning Time

We compare the tuning time of the proposed DAIM and the other indexing schemes, changing *R_hot_, R_qw_*, the bucket size, *n*, and *H_r_*.

**Figure 11.**
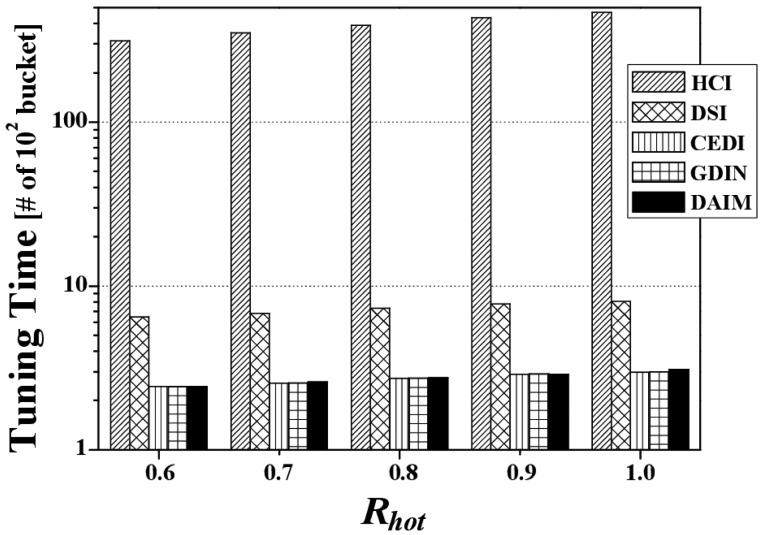
The comparison of the tuning time over various *R_hot_*.

Figure 11 discloses the effect of changes in *R_hot_* on the tuning time. Figure 11 depicts the tuning time for both schemes according to various values of *R_hot_* at *R_qw_* = 0.06 with a bucket size of 64 bytes. The tuning times for DAIM, CEDI, and GDIN are much shorter than HCI and DSI. That is because HCI and DSI let the clients extract the results of a given query after listening to too many candidates, as chosen through HC values, not through real coordinates, whereas CEDI, GDIN, and DAIM allow the clients to only listen to their desired data items by extracting them using the real coordinates, avoiding redundant listening. The tuning time for DAIM is almost the same as CEDI and GDIN because all the schemes let the clients listen to only the queried items, avoiding any extra data items. When *R_hot_* is 1.0, the tuning time of DAIM is slightly longer than that of GDIN. It is because DAIM makes the clients access *MI* for downloading hot data items from the channel in addition to *CI*, differently from *GDIN* that does not keep MaxBRs for hot data items.

**Figure 12.**
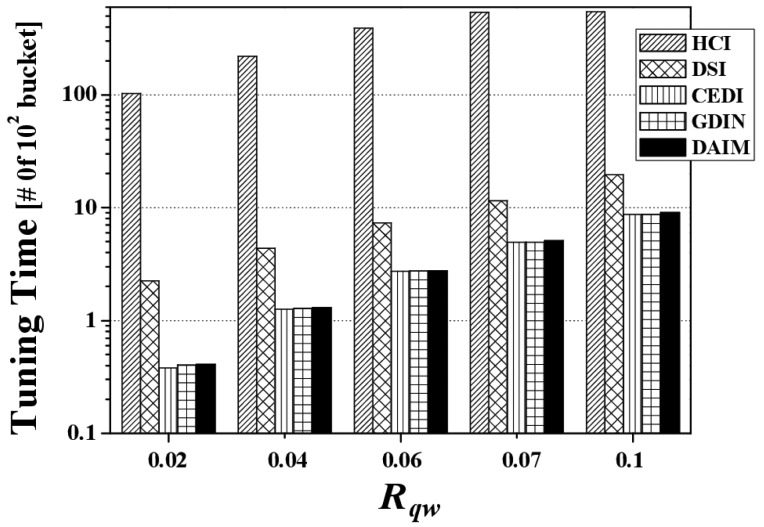
The comparison of the tuning time over various *Rqw*.

Figure 12 reveals the effects of changing *R_qw_* on the tuning time. Figure 12 shows the tuning time of the schemes according to various values of *R_qw_* at *R_hot_* = 0.8 with a bucket size of 64 bytes. As the query size increases, the tuning time increases in all indexing schemes. That is because, the larger queries are, the more data items they contain. DAIM, CEDI, and GDIN outperform HCI and DSI owing to query processing based on real coordinates, as opposed to using HC values, as in HCI and DSI. In the figure, the slightly larger tuning time of DAIM than that of GDIN at *R_qw_* = 0.1 results from the reason mentioned in the analysis of Figure 11.

**Figure 13.**
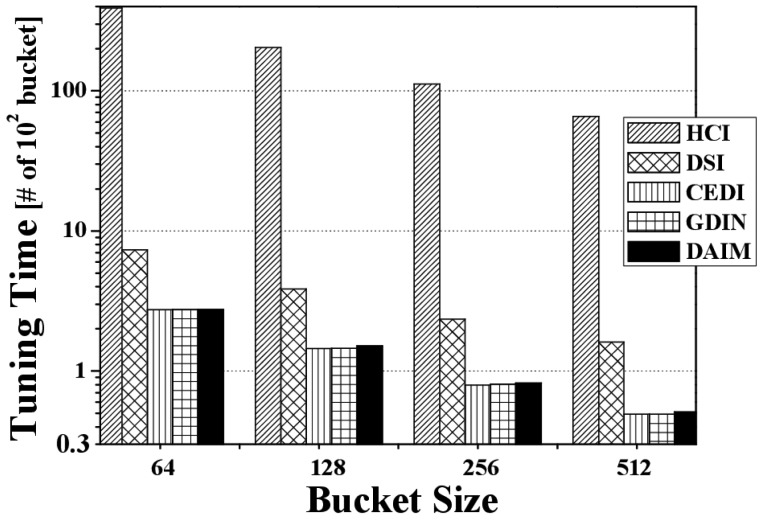
The comparison of the tuning time over various bucket sizes.

Figure 13 discloses the effect of changing the bucket size on the tuning time. Figure 13 depicts the tuning time over various bucket sizes at *R_qw_* = 0.06 and *R_hot_* = 0.8. We measure the tuning time in buckets because the bucket is the smallest logical unit in which the clients access data items. As the bucket size increases, the tuning time decreases. That is because as the bucket size increases, the number of buckets decreases, as seen with the access time. In the figure, the slightly larger tuning time of DAIM than that of GDIN at various bucket sizes results from the reason mentioned in the analysis of Figure 11.

**Figure 14.**
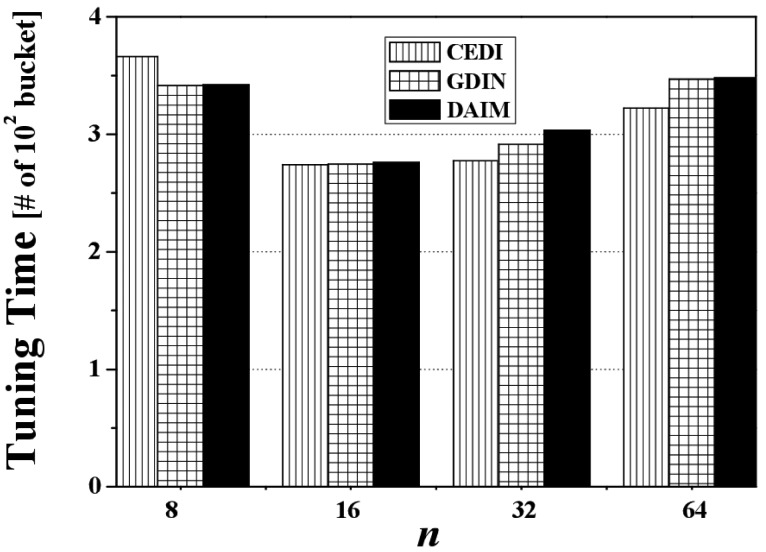
The comparison of the tuning time over various *n*.

Figure 14 reveals the effect of changing *n*, the grid partition, on the tuning time for CEDI, GDIN, and DAIM when *R_qw_* and *R_hot_* are 0.06 and 0.8, respectively. Figure 14 demonstrates that the tuning time under DAIM is almost the same as GDIN and CEDI for various values of *n*, as mentioned previously. In the figure, the larger tuning time of DAIM than that of GDIN at *n* = 32 results from the reason mentioned in the analysis of Figure 11.

Varying *n* affects the tuning time by the number of index tables to access and the size of an index table. At smaller *n*, the size of an index table is larger than that at larger *n*. It is because the number of data items included in a cell increases, as decreasing the value of *n*. As increasing the value of *n*, the number of index tables to access increases. It is because the number of cells overlapped with a given query window increases. Accordingly, as decreasing the value of *n*, the tuning time increases by the size of an index table. Also, as increasing the value of *n*, the tuning time increases by the number of index tables to access.

**Figure 15.**
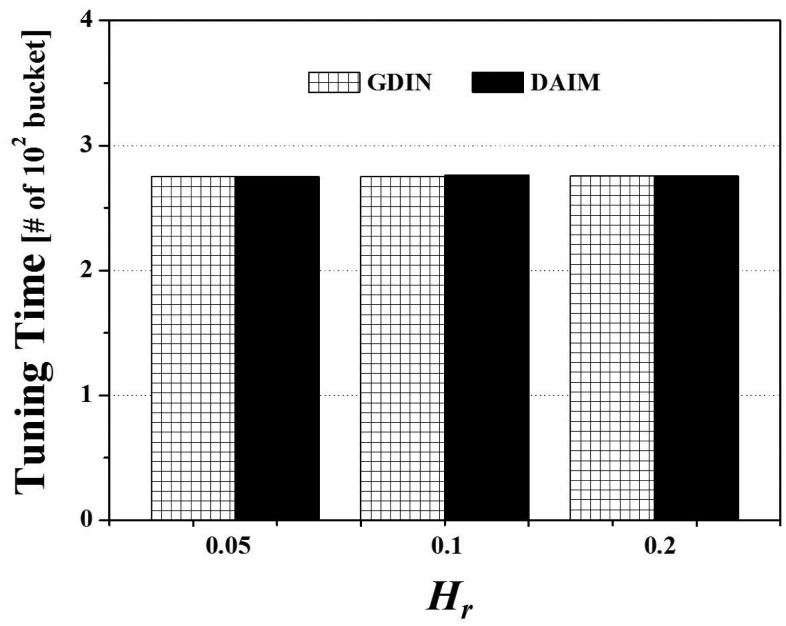
The comparison of the tuning time over various *H_r_*.

Finally, we consider the effect of the different distribution of the access probabilities of data items on the tuning time. Figure 15 depicts the effects of the change of *H_r_*, the ratio of hot data items, on the tuning time for the two non-flat broadcasting schemes, GDIN, and DAIM, when *R_qw_* and *R_hot_* are 0.06 and 0.8, respectively. Figure 15 reveals that the tuning time of DAIM is almost same with that of GDIN over various values of *H_r_*. The tuning times of GDIN and DAIM remain unchanged as other parameters keep constant. It is because *H_r_* affects the length of the broadcast cycle by changing the number of hot data items and does not affect the number of data items that the clients have to access to process the given queries. The tuning time is affected by the parameters that decide the number of data items and index tables to access, like *R_qw_* and *R_hot_*.

### 4.4. Comparison of the Consumed Energy

To show how practical DAIM is, we compare the energy consumption of the clients while processing the given queries. We measure the energy consumption, a performance metric combining the access time and tuning time, with the sum of the energy consumed in the active and the doze modes.

In order to evaluate the amount of energy consumed, we consider two components of a client, the CPU and the NIC(Network Interface Card). Table 1 shows *ε_active_* and *ε_doze_*, the amount of energy that the CPU and the NIC consume in the active mode and the doze mode per second, respectively. As can be seen in the table, the client consumes 25.16 mW/s in the doze mode and 1150 mW/s in the active mode [27].

The entire amount of energy, *E_q_*, consumed during processing given queries is modeled with *ε_active_* and *ε_doze_* by the following equation:

(6) $$E_{q}=\varepsilon_{\text{active}}T_{\text{tuning}}+\varepsilon_{\text{doze}}\left(T_{\text{access}}-T_{\text{tunning}}\right)$$

Here, *T_tuning_* is the tuning time of the clients which is the amount of time the clients operate in the active mode listening to the channel in order to download queried data items. *T_access_* is the access time of the clients. The term (*T_tuning_* − *T_access_*) represents the amount of time that the clients operate in the doze mode [27].

**Figure 16.**
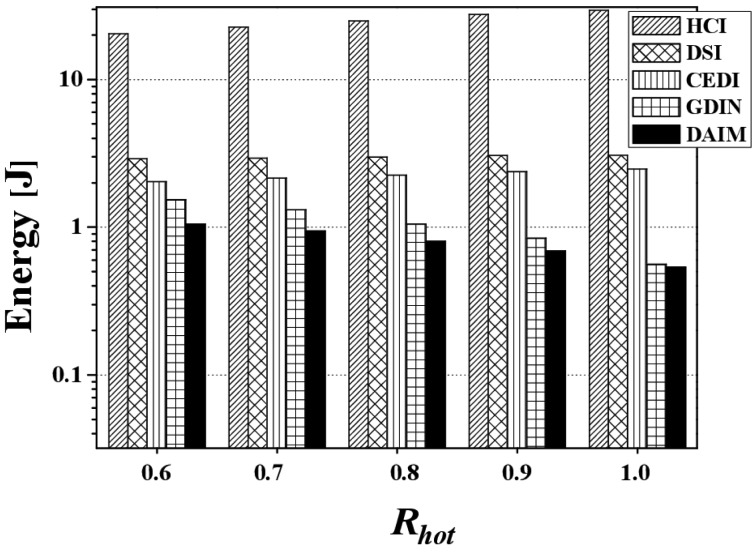
The comparison of the energy consumption over various *R_hot_*.

Figure 16 shows the energy consumption from changing *R_hot_*. Figure 16 depicts the energy consumption *E_q_* by changing *R_hot_* at *R_qw_* = 0.06 with a bucket size of 64 bytes. The figure reveals that DAIM lets the clients utilize a significantly lower amount of energy for given queries, compared to other index schemes. The trend for energy consumption follows that of the access time according to various values of *R_hot_* with a fixed *R_qw_* and bucket size. The reason for this is that the access time is much longer than the tuning time, meaning that the time duration in the doze mode is much longer than the time duration in the active mode, despite having far lower energy consumption in the doze mode. The much longer time duration in that mode makes the total amount of energy consumption larger than the active mode. Although DAIM shows almost the same tuning time as GDIN, *E_q_* of DAIM is about 76% that of GDIN at *R_hot_* = 0.8 and *R_qw_* = 0.06. This results from the shorter access time compared to GDIN. With respect to practical implications, Figure 16 reveals how important it is to reduce the access time.

**Figure 17.**
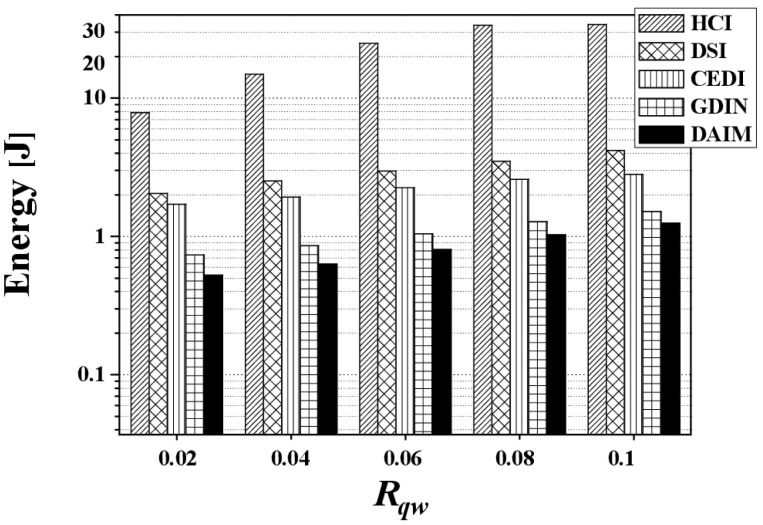
The comparison of the energy consumption over various *R_qw_*.

Figure 17 discloses the energy consumption from changing *R_qw_*. Figure 17 shows the energy consumption *E_q_* by changing *R_qw_* at *R_hot_* = 0.8 with a bucket size of 64 bytes and *n* = 16. The figure demonstrates that DAIM helps the clients process given queries in an energy efficient manner for all query sizes, compared to other indexing schemes. As the query size increases, the energy consumption increases. This is a result of the increase in the access time and tuning time according to increases in query size.

**Figure 18.**
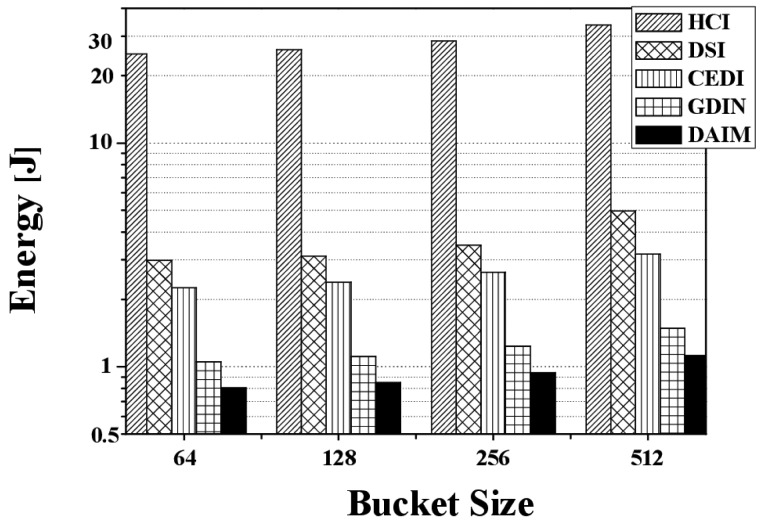
The comparison of the energy consumption over various bucket sizes.

Figure 18 reveals the energy consumption from changing the bucket size. Figure 18 depicts energy consumption over various bucket sizes at *R_qw_* = 0.06, *R_hot_* = 0.8, and *n* = 16. With the various bucket sizes, DAIM allows the clients to process the given window queries efficiently with respect to energy consumption. In the figure, the energy consumption increases as the bucket size increases, unlike the access time and the tuning time. That results from that the access time and the tuning time in buckets are converted to bytes in order to calculate the energy consumption. The access time and the tuning time converted to bytes increase as the bucket size increases. It is because the bucket header, that keeps the time pointer to the next index table, is added to a bucket when a data item is organized into buckets. For example, if we consider the bucket header of 8 bytes and a bucket of 64 bytes, a data item of 1024 bytes is organized into 19 buckets. In the example, a client has to access 19 buckets, *i.e.*, 1216 bytes, in order to download the data items from the channel. When we consider the same bucket header and a bucket of 512 byte, the data item of 1024 bytes is organized into 3 buckets. The client have to access 3 buckets, *i.e.*, 1536 bytes, for downloading the data item. Thus, as the size of the bucket increase, the access time and the tuning time increase in bytes, while decrease in buckets.

**Figure 19.**
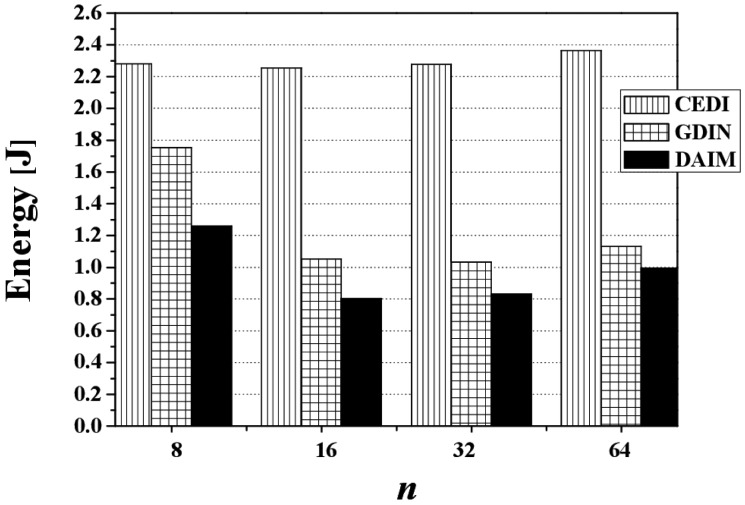
The comparison of the energy consumption over various *n*.

Figure 19 discloses the effect of changing *n*, the grid partition, on energy consumption for CEDI, GDIN, and DAIM when *R_qw_* = 0.06 and *R_hot_* = 0.8 with a bucket size of 64 bytes. Figure 19 shows that DAIM outperforms CEDI and GDIN in energy consumption with various partitions of data space. This results from the reduced access time despite the almost same tuning time under DAIM, compared to CEDI and GDIN.

**Figure 20.**
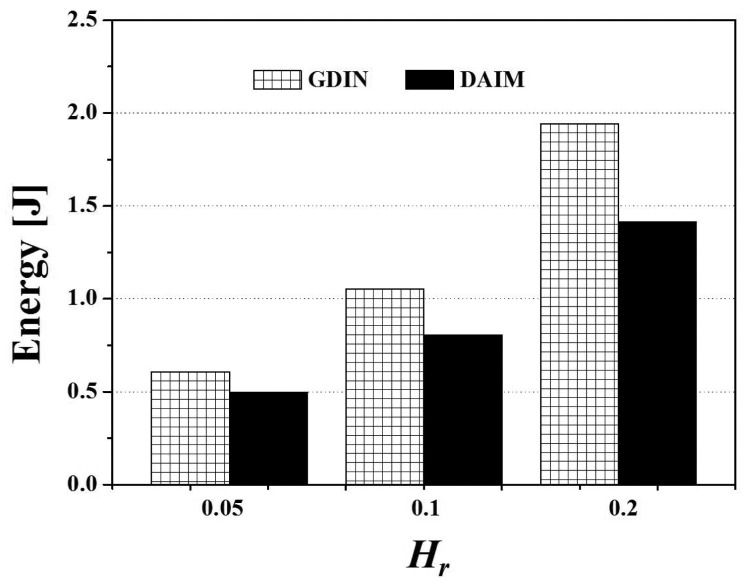
The comparison of the energy consumption over various *H_r_*.

Lastly, Figure 20 shows the effect of the different distribution of the access probabilities of data items on the energy consumption. The figure depicts the results of the change of *H_r_*, the ratio of hot data items, for the two non-flat broadcasting schemes, GDIN, and DAIM, when *R_qw_* and *R_hot_* are 0.06 and 0.8, respectively. In Figure 20, DAIM outperforms GDIN over various values of *H_r_*. It mainly results from that DAIM shows shorter access time than GDIN, despite of almost same tuning time of DAIM with that of GDIN.

## 5. Conclusions

We have described spatial indexes and processing window queries in the non-flat wireless spatial data broadcasting. The proposed DAIM provides the clients with a way to quickly access desired data items when their data access patterns are weighted towards so-called hot items. DAIM adopts a MaxBR to separate hot items from regular items. Using MaxBRs over a data space, only hot items are replicated multiple times in a broadcast cycle, unlike GDIN (the existing scheme in non-flat broadcasting). This means DAIM improves the access time and reduces energy consumption while processing the given queries. Through simulations, we show that DAIM outperforms the existing indexing scheme, GDIN, in non-flat broadcasting, as well as HCI, DSI, and CEDI in flat broadcasting, over various clients' data access patterns and sizes of queries. In the future, we will research continuous window query processing on air.

## Figures and Tables

**Table 1.** Energy consumption of a client (in *mW/s*).

	**Model**	*ε_doze_*	*ε_active_*
CPU	StrongARM SA-1100	0.16	400
NIC	RangeLAN2 7401/02	25	750
